# Influence of Alcohol

**Published:** 1877-10

**Authors:** 


### Influence of Alcohol.

General Paresis.—By facts ascertained regarding patients in asylums, the belief as to the influence of alcoholic intemperance as an active cause, is strongly sustained. In one hundred and fifty-five patient, one hundred and sixteen gave a history of habitual intemperance, while all but ten claim the designation of moderate drinkers. . . . The question arises whether insanity does not precede the drunkenness or lead to it, and not the reverse order of procedure.

Dr. McDonald, *American Journal of Insanity.*

